# Virulence Characterization of *Salmonella enterica* by a New Microarray: Detection and Evaluation of the Cytolethal Distending Toxin Gene Activity in the Unusual Host *S*. Typhimurium

**DOI:** 10.1371/journal.pone.0135010

**Published:** 2015-08-05

**Authors:** Rui Figueiredo, Roderick Card, Carla Nunes, Manal AbuOun, Mary C. Bagnall, Javier Nunez, Nuno Mendonça, Muna F. Anjum, Gabriela Jorge da Silva

**Affiliations:** 1 Faculty of Pharmacy and Center for Neuroscience and Cell Biology, University of Coimbra, Coimbra, Portugal; 2 Department of Bacteriology, Animal and Plant Health Agency, Weybridge, New Haw, Addlestone, Surrey, KT15 3NB, United Kingdom; 3 Specialist Scientific Support, Animal and Plant Health Agency, Weybridge, New Haw, Addlestone, Surrey, KT15 3NB, United Kingdom; Institut National de la Recherche Agronomique, FRANCE

## Abstract

*Salmonella enterica* is a zoonotic foodborne pathogen that causes acute gastroenteritis in humans. We assessed the virulence potential of one-hundred and six *Salmonella* strains isolated from food animals and products. A high through-put virulence genes microarray demonstrated *Salmonella* Pathogenicity Islands (SPI) and adherence genes were highly conserved, while prophages and virulence plasmid genes were variably present. Isolates were grouped by serotype, and virulence plasmids separated *S*. Typhimurium in two clusters. Atypical microarray results lead to whole genome sequencing (WGS) of *S*. Infantis Sal147, which identified deletion of thirty-eight SPI-1 genes. Sal147 was unable to invade HeLa cells and showed reduced mortality in *Galleria mellonella* infection model, in comparison to a SPI-1 harbouring *S*. Infantis. Microarray and WGS of *S*. Typhimurium Sal199, established for the first time in *S*. Typhimurium presence of *cdtB* and other Typhi-related genes. Characterization of Sal199 showed *cdtB* genes were upstream of transposase *IS*911, and co-expressed with other Typhi-related genes. Cell cycle arrest, cytoplasmic distension, and nuclear enlargement were detected in HeLa cells infected by Sal199, but not with *S*. Typhimurium LT2. Increased mortality of *Galleria* was detected on infection with Sal199 compared to LT2. Thus, *Salmonella* isolates were rapidly characterized using a high through-put microarray; helping to identify unusual virulence features which were corroborated by further characterisation. This work demonstrates that the use of suitable screening methods for *Salmonella* virulence can help assess the potential risk associated with certain *Salmonella* to humans. Incorporation of such methodology into surveillance could help reduce the risk of emergence of epidemic *Salmonella* strains.

## Introduction

Foodborne diseases caused by non-typhoidal *Salmonella* (NTS) represent an important public health problem and economic burden worldwide [1]. In Europe, there are over 100,000 salmonellosis human cases each year [2]. *Salmonella* serovars associated with gastroenteritis transmit through the faecal-oral route, either directly or via contaminated food or water [3]. Gastroenteritis infections caused by NTS are mostly self-limiting and antimicrobial treatment is usually not required. However, 3–10% of individuals with gastrointestinal illness caused by NTS will develop bacteraemia [4], a serious and potentially fatal condition that requires antimicrobial treatment [3]. The increase in bacterial antimicrobial resistance has led to an increased risk of treatment failure in humans and reduces significantly the therapeutic options available [2]. While a large number of *Salmonella* serotypes can cause gastroenteritis, a few serotypes such as *S*. Typhi can cause invasive infection.

The differences in pathogenicity of *Salmonella* serotypes depend upon the virulence potential of the microorganism and the susceptibility of the host. Bacterial virulence factors are necessary to adhere, invade and replicate inside host cells. These are encoded by genes present on a wide range of genetic elements, including the bacterial chromosome, plasmids, prophages and *Salmonella* Pathogenicity Islands (SPIs). Some SPIs are conserved throughout the genus, e.g. SPI-1, which encodes a Type 3 Secretion System 1 (T3SS-1) which is important for infection of the host cell and SPI-2, which encodes T3SS-2 that facilitates intracellular survival and replication [5]. Other SPIs are serotype specific *e*.*g*. SPI-7 in *S*. Typhi [6], and increase the virulence potential of the pathogen. Twenty-percent of the *Salmonella* genome comprises DNA of bacteriophage origin which encodes a variety of virulence factors [7]. Plasmids are frequently found in *Salmonella* serotypes associated with infections of humans and animals, including the *Salmonella* virulence plasmid. In addition to the risk presented by NTS serotypes as a potential foodborne hazard, there has been a rapid emergence in recent years of multidrug-resistant isolates of *Salmonella* [8, 9]. So, it is relevant to investigate the presence of virulence determinants in strains that have been implicated in *Salmonella* disease of food origin to help assess and understand the risk posed by strains from different sources. High through-put DNA microarrays have been developed which enable detection of large numbers of virulence genes, allowing many isolates to be studied simultaneously [10–13]. This chip is unique as it is based on detecting virulence determinants and not serotype, unlike the other *Salmonella* high through-put chips [11; 12], which makes this chip much more informative to determine the potential risk posed by these isolates. It is also much cheaper and easier to handle unlike other *Salmonella* glass-slide microarray chips which have been used to look at virulence profile previously [10; 14; 15].

The aim of the present study was to assess the diversity of virulence genes in *S*. *enterica* in order to better understand the potential risk that they might present. A customised *Salmonella* virulence gene microarray was constructed targeting *Salmonella* pathogenicity islands, virulence regulators, prophages, toxin, and virulence plasmids genes. The array screening informed the selection of atypical isolates for further *in vitro* and i*n vivo* virulence characterisation using whole genome sequencing, adhesion/invasion assays, *Galleria mellonella* infection model, and flow cytometry.

## Methods

### *Salmonella* strains

A total of 106 *Salmonella* strains were selected from a previous epidemiological study [16]. Strains had been isolated from poultry (n = 39), swine (n = 14), cattle (n = 1), and processed food (meat cuts, hamburgers or sausages) (n = 52). The serotypes found were Typhimurium (n = 59), Enteritidis (n = 21), Havana (n = 6), Infantis (n = 6), Mbandaka (n = 2), Virchow (n = 2), Derby (n = 1) and non-determined serotype (n = 9) (Table 1).

**Table 1:** Frequency of non-typhoidal *Salmonella* serotypes according origin.

		Poultry (n = 39) Nr (%)			Swine (n = 14)^a^ Nr (%)		Cattle (n = 1)^b^ Nr (%)	Processed food of animal origin Nr (%)	
*Salmonella* Serotype	Total (n = 106) Nr (%)	Farm	Slaughter house	Distribution	Slaughter house	Distribution	Slaughter house	Poultry (n = 21)	Swine (n = 31)
Typhimurium	59 (55.7)	2 (5.1)	6 (15.4)	-	10 (71.4)	1 (7.1)	1 (100)	14 (66.6)	25 (80.6)
Enteritidis	21 (19.8)	9 (23.1)	-	9 (23.1)	-	-	-	3 (14.3)	-
Infantis	6 (5.7)	-	1 (2.6)	-	1 (7.1)	-	-	-	4 (12.9)
Havana	6 (5.7)	6 (15.4)	-	-	-	-	-	-	-
Mbandaka	2 (1.9)	2 (5)	-	-	-	-	-	-	-
Virchow	2 (1.9)	2 (5)	-	-	-	-	-	-	-
Derby	1 (0.9)	1 (2.6)	-	-	-	-	-	-	-
ND^c^	9 (8.4)	1 (2.6)	-	-	2 (14.2)	-	-	4 (19)	2 (6.5)

^a^Any isolates recovered from Farm

^b^Any isolate recovered from farm and distribution.

^c^Not determined

### Design and validation of *Salmonella* virulence gene microarray probes

A total of 114 *Salmonella* virulence determinants (S1 Table) present on the chromosome (n = 90; SPIs, n = 52, and other chromosomal genes, n = 38), phage related (n = 12), and plasmid related (n = 12), were selected. The genes span a range of virulence and regulatory associated functions including adhesion (n = 28), toxins (n = 2), regulation (n = 2), and invasion/survival within non-phagocytic enterocytes (n = 82). The hybridisation probes and labelling primer sequences were designed in this study and their specificity tested *in silico* as previously described [17]; the sequences are presented in S1 Table. In brief, sequence specific primers for the labelling reaction and hybridisation probes were designed based on sequence data available in GenBank. First, a sequence entry was retrieved for each target gene in the gene list (S1 Table), as a reference. Then BLASTn searches against the whole GenBank (NR database) were performed to find any further entries similar to the reference sequence. This was performed for every target gene. The hits for each target were aligned. The alignments were generated directly from the BLASTn results and used to identify conserved regions. Probes and primers were designed, if possible, within these regions. If no sufficient consensus site was found, pairs of perfect match/mismatch primers or probes were designed which were specific for the allelic variants. The size of the oligonucleotides was varied so the melting temperatures were nearly identical for all probes and primers. A total of 117 gene probes and 114 labelling primers were designed for these genes, including four probes being designed for one gene to encompass allelic variations. Specificity of all probes and primers were determined by performing BLASTn searches against the NCBI database. Each oligonucleotide probe was spotted in duplicate and the microarrays printed by Alere Technologies (Jena, Germany); each replicate probes were spotted at different positions to circumvent any spatial effect of hybridization. The performance of the probes was assessed using control strains, in which presence of the gene had been verified by PCR and sequencing (see below). The specificity of each probe was estimated by comparing the microarray result with PCR and sequence data for the control panel of strains.

### Microarray Hybridization

Genomic DNA was isolated and used for a linear multiplex amplification reaction which was performed at 55°C, as previously described [10]. The PCR mixture for linear DNA amplification and labelling contained DNA (0.5μg) in a total volume of 10 μl, mixed with 1μl of 10x Therminator amplification buffer, 0.1μl Therminator DNA polymerase (BioLabs), 1μl of virulence primer-mix (0.135 μM per oligonucleotide in the stock solution), 1μl of dNTP-mix including the biotin label (1mM dACGTP; 0.65mM dTTP) and 0.35μl Biotin-16-dUTP (1 mM Biotin-16-dUTP, Roche). Hybridization was achieved using the HybridisationPlusKit (Alere Technologies, Jena, Germany) employing a heated mixer (Thermomixer, Eppendorf, Hamburg, Germany). Single-stranded labelled amplified products were hybridised to the arrays as described previously [18] and signals read on an ArrayMate apparatus (Alere Technologies, Jena, Germany) using IconoClust software (standard version; Inverness Technologies, Jena, Germany) [17].

### Microarray data analysis and probes validation

Mean signal intensities of two replicate spots per probe were used for analysis. Based on PCR data, for the virulence gene probes intensities ≥ 0.4 were considered positive, while those with value < 0.4 were considered absent. The microarray data across all isolates were converted to a binary format, whereby 1 indicated gene presence and 0 indicated gene absence, and compared in BioNumerics 5.1 (Applied Maths, Sint-Martens-Latem, Belgium). An Unweighted Pair Group Method with Arithmetic mean (UPGMA) dendrogram was used to cluster isolates based on their virulence determinants.

PCR primers for each virulence gene were designed using the Primer3Web software (version 0.4.0) (http://frodo.wi.mit.edu/) using the reference sequences given in S1 Table, to give a 200 to 500 bp amplicons. PCR amplifications were performed using standard conditions, as reported previously [17]. A control *Salmonella* strain was identified for each virulence gene (S1 Table) and presence of the gene confirmed by PCR and sequencing.

### Whole Genome Sequencing

The genomic DNA from selected isolates showing atypical microarray results was pair-end sequenced using an Illumina Miseq Sequencer. Raw sequences were filtered and trimmed to minimized sequencing errors (Trimmomatic software) [19]. The raw data was mapped onto the reference *S*. Typhimurium LT2 (BWA software) [20] and SAMTOOLS software [21]. Between 85% and 91% of the raw data was mapped to LT2 with a mean coverage parameter between 17 and 33. Between 89% and 97% of the reference genome LT2 was shared with the strains used in this study. In house software was used to identify the core genome (genomic regions shared by the strains in the study), SNPs, possible deletions (uncovered regions of the reference genome LT2) and insertions (de novo assembly of unmapped raw data) with respect to LT2 strain (Accession number: AE006468). The sequences of the *S*. Typhimurium Sal199, *S*. Infantis Sal147, and *S*. Infantis Sal280 are deposited in the European Nucleotide Archive (ENA) under the study accession number PRJEB9808, which is available from http://www.ebi.ac.uk/ena/data/view/PRJEB9808.

### Reverse transcriptase PCR of SPI-11 genes and typhoid-associated virulence genes

Total RNA was isolated from *S*. Typhimurium Sal199 and *S*. Typhimurium LT2 strains grown on Mueller-Hinton broth for 2, 16 and 24 h at 37°C by using Tri reagent (Sigma Aldrich, Poole, UK) as described before [22]. Reverse transcriptase PCR (RT-PCR) was carried out using the OneStep RT-PCR kit (Quiagen, UK), according to the manufacturer's instructions. RT-PCR was performed using primers designed to SPI-11 genes *envF*, *pagC*, *pagD*, *pltA*, *pltB*, *cdtB* and three typhoid-related virulence genes *taiA*, *tcfA* and *hlyE* (S2 Table). Genomic DNA and *agfA*, a fimbrial gene, were used as positive controls, in normal PCR and RT-PCR, respectively. A negative control with no DNA was also included in the assays. RT-PCR was performed with an initial incubation step of 50°C for 30 min, during which time cDNA was synthesized from the RNA template. The reverse transcriptase was inactivated and the cDNA denatured by incubation at 95°C for 15 min, followed by an amplification reaction comprising 30 cycles of 94°C for 1 min, 55°C for 0.5 min, and 72°C for 2 min. RT-PCR products were visualized on 1% (w/v) agarose gels.

### Cell distending assays and flow cytometry

Cytolethal toxin assays for *S*. Typhimurium Sal199 strain were performed as described previously by Spanó *et al*. [23] with minor modification using *S*. Typhimurium LT2 as negative control. Briefly, HeLa cells were seeded into 6-well tissue culture plates at a density of 1×10^5^ cells per well. The cells were then incubated for 72 h at 37°C with 5% CO_2_. After the incubation, cells were washed three times with Phosphate-Buffered Saline 0.1M, pH 7.2 (PBS), fixed with 3% paraformaldehyde, and observed under light microscope Axiovert 40 CFL (Carl Zeiss Microscopy, LLC, United States), for demonstration of morphological changes in the cells. To measure the cell cycle arrest flow cytometry was followed as described by Mezal *et al*. [24]. The cells’ pellets were resuspended with propidium iodide (PI) staining solution (0.1% Triton X-100, 40 μg of PI mL^-1^, and 100 μg of RNase A mL^-1^) and incubated at room temperature in the dark for 15 min. Approximately, 1.0×10^4^ cells were examined using a Fluorescence-activated cell sorting (FACS) analysis with the excitation set at 488 nm and the emission set at 630 nm. The experiment was conducted in triplicate.

### Adhesion/invasion assay

Two *S*. Infantis isolates, Sal147 missing 14 SPI-1 genes, and Sal280 with all SPI-1 genes were selected. Adhesion and invasion assays were conducted using HT-29 colon human cells in presence of gentamicin as previously described [25]. Mean of the counts from each well for the four replicates were log_10_ transformed to equalise the variances for each strain regardless of the mean. The resulting four means for adhesion and four means for invasion were subjected to a two-ways analysis of variance (ANOVA) using the statistical package GraphPad Prism 5 (GraphPad Software, Inc., California, USA). A *p*-value of < 0.05 was taken to indicate statistical significance.

### *Galleria mellonella* Infection model

*G*. *mellonella* was used as an *in vivo* virulence model, as previously described [26] to compare the *in vivo* virulence of Sal199, Sal147, and Sal280, in comparison with to *S*. Typhimurium LT2. Briefly, *Salmonella* grown in Mueller-Hinton broth for 2, 16 and 24h at 37°C, was harvested to give a final concentration of 10^8^ bacteria in 1 mL 0.1 M PBS (pH 7.2). Ten larvae were injected with a 10 μL 10^4^ CFU mL^-1^ in the right fore pro-leg, using a Hamilton syringe (Sigma, UK). All larvae were incubated at 37°C for 24 h before determining rates of survival and macroscopic appearance. Non-injected and 0.1 M PBS-injected animals were included in each experiment as controls. The exact dose was determined after plating the inoculum on LB agar. Representative results were obtained from three independent experiments. Data are expressed as percentage of survival, and analysed using the statistical package GraphPad Prism 5 (GraphPad Software, Inc., California, USA). A *p*-value of < 0.05 was taken to indicate statistical significance.

## Results

### Virulence genes detected in isolates

**Fig 1:**
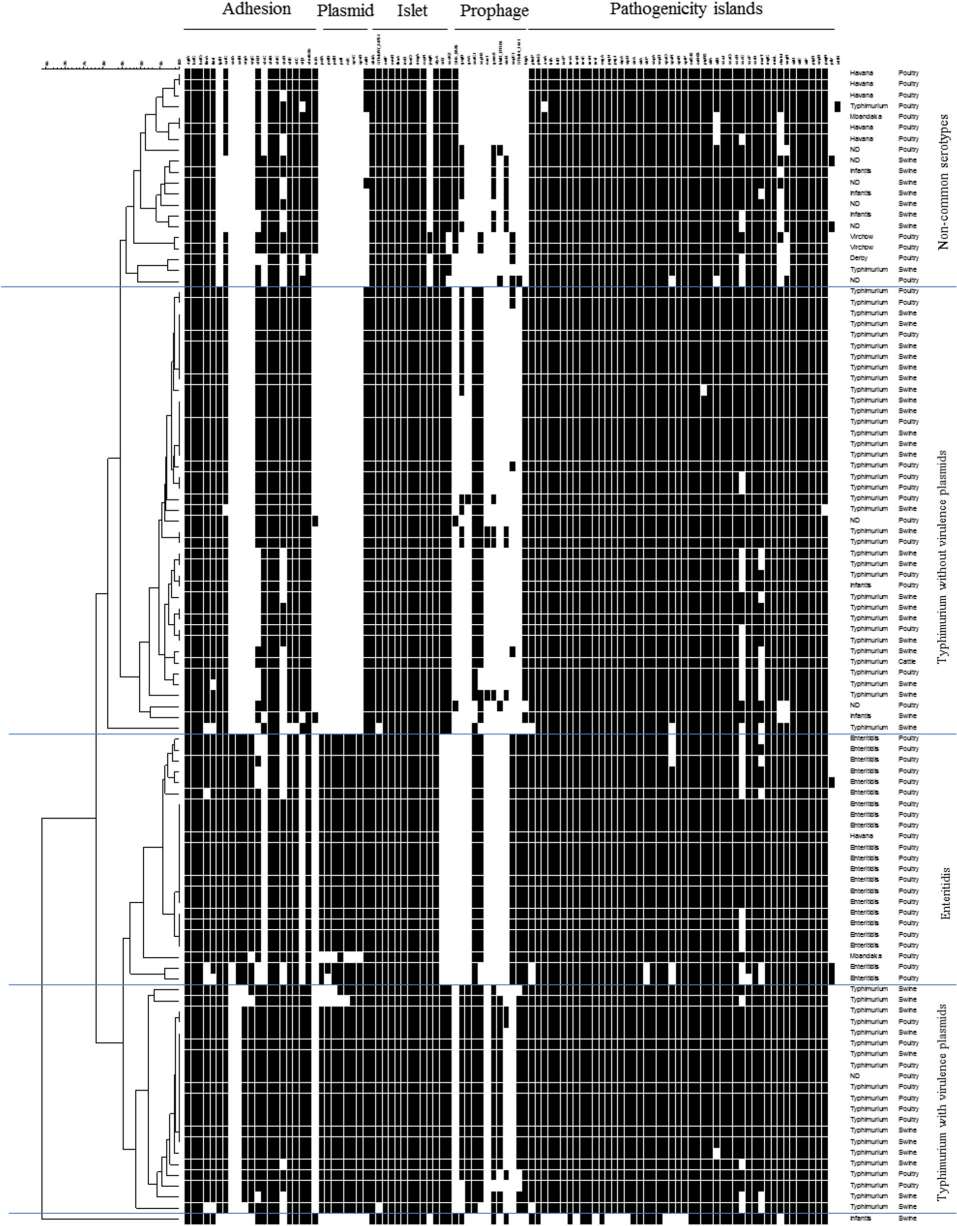
Virulence determinants microarrays data for 106 *Salmonella* strains analysed. At the top, the analysed genes are grouped according to their particular genomic location or function (fimbrial). The order of strains represents their relatedness according to the UPGMA dendrogram type performed in BioNumerics 5.1. The hybridization result of a distinct strain is shown by row. A white box indicates the absence and a black box indicates the presence of the target sequence in the strain.

One hundred and eight probes and primers designed in this study for 105 genes were validated with control strains (S1 Table). Nine genes could not be validated as the probes were non-specific and have not been considered any further (data not shown). Microarray was performed on the aforementioned 106 *Salmonella* isolates. The microarray results were transformed to a binary form *i*.*e*. present or absent (S3 Table) and analysed in BioNumerics by an UPGMA cluster analysis (Fig 1). Cluster analysis showed four different clusters: one contained 20 isolates which were mainly from the non-common serotypes; one contained all *S*. Enteritidis isolates and there were two clusters of *S*. Typhimurium, one with 40 isolates without virulence plasmid and prophage associated genes and another comprising 20 isolates with these determinants (Fig 1).

The results indicated that virulence determinants located in SPIs 1–6 were highly conserved (Fig 1). In most isolates all SPI-1 genes selected for the microarray were present. One strain of *S*. Infantis (Sal147) isolated from a food product of swine origin was deficient in many SPI-1 genes. Sixteen isolates of non-common serotypes, lacked *rhuM* (hypothetical DNA-binding protein), and seven isolates lacked *sugR* (hypothetical ATP-binding protein) from SPI-3. Virulence determinants of SPI-4 and SPI-5 were conserved in all isolates. Some islet genes were present in all serotypes (Fig 1). The islet group was highly conserved genes and included those encoding regulator (*envR*, *fhuA*, *oxyR*, *entF*, *slyA*, *leuO*, *msgA*) or effector proteins (*sseK2*, *sfrJ*, *iroB* and *pagK*).

The prophage genes showed the greatest variation in their occurrence (Fig 1). Gifsy-1-associated gene *gogB* were present in half of *S*. Typhimurium and *S*. Infantis isolates. The Gifsy-2-associated gene *sodCI* was detected in 100% of *S*. Enteritidis and 96.6% strains from *S*. Typhimurium and the non-common serotypes. The Gifsy-3-associated gene *sspH2* was reported in 89.8% strains of *S*. Typhimurium, and 90.5% of *S*. Enteritidis.

The genes *spvC* and *spvR* are located on the *Salmonella* virulence plasmid. They were present in all isolates of *S*. Enteritidis and in 33.9% of *S*. Typhimurium. Gene *rck* was detected in all *S*. Enteritidis, and in 32.2% *S*. Typhimurium isolates.

The fimbrial genes *agfA*, *bcfC*, *bcfG*, *stdB*, *stiC*, STM4595 were present in all isolates. The *sefA*/*sefR* was found in 90.5% *S*. Enteritidis. All *S*. Enteritidis isolates had the plasmid-located genes (*rck*, *pefA*, *pefD*, *pefI*, *spvC*, *spvR*, *srgA*, and *srgC*). Typhi colonization factor (*tcf*) operon was found in 18.9% of isolates from serotypes Typhimurium, Mbandaka, Havana, Virchow, and Infantis. However, the presence of pilus-fimbrial *pilV* gene was only found in three isolates of non-determined serotype.

### Whole Genome Sequencing data

Whole genome sequencing (WGS) was performed on two isolates which showed atypical microarray results; Sal199 which showed presence of the *cdtB* gene by array and Sal147 which showed absence of SPI-1 genes included on the array. The WGS data was analysed by mapping contigs to the *S*. Typhimurium LT2 genome, which was used as reference. In *S*. Typhimurium Sal199, the insertion of *cdtB* toxin together with *pltA* and *pltB*, was positioned upstream of the transposase *IS*911. Analysis of *S*. Infantis Sal147 genome, corroborated arrays results showing deletion of a large number of SPI-1 genes, namely *avrA*, *sprB*, *hilC*, *orgC*, *orgB*, *orgA*, *prgK*, *prgJ*, *prgI*, *prgH*, *hilD*, *hilA*, *iagB*, *sptP*, *sicP*, *iacP*, *sipA*, *sipD*, *sipC*, *sipB*, *sicA*, *spaS*, *spaR*, *spaQ*, *spaP*, *spaO*, *invJ*, *invI*, *invC*, *invB*, *invA*, *invE*, *invG*, *invF*. Overall, both genomes were very elastic with many phages, and mobile elements being inserted or deleted, with respect to LT2.

### SPI-11 genes and typhoid-related virulence genes

Arrays and WGS data indicated the unusual presence of *cdtB* gene on *S*. Typhimurium Sal199. The *cdtB* gene is encoded on SPI-11, together with *pltA* and *pltB*; when these genes are expressed together they form a tripartite toxin, which was originally described in *S*. Typhi [23]. In order to check the expression of these three genes, and *taiA*, *tcfA*, and *hlyE* which are other typhoid associated genes, RT-PCR was performed. The RT-PCR experiments showed the expression of SPI-11 genes, as well as the expression of typhoid-related virulence genes (S2 Table). *cdtB* gene as well as other SPI-11 genes were detected on *S*. Typhimurium Sal199, and some SPI-11 genes were also present in LT2, but not the *cdtB* toxin genes (S2 Table).

### Toxin cells assays and flow cytometry

**Fig 2:**
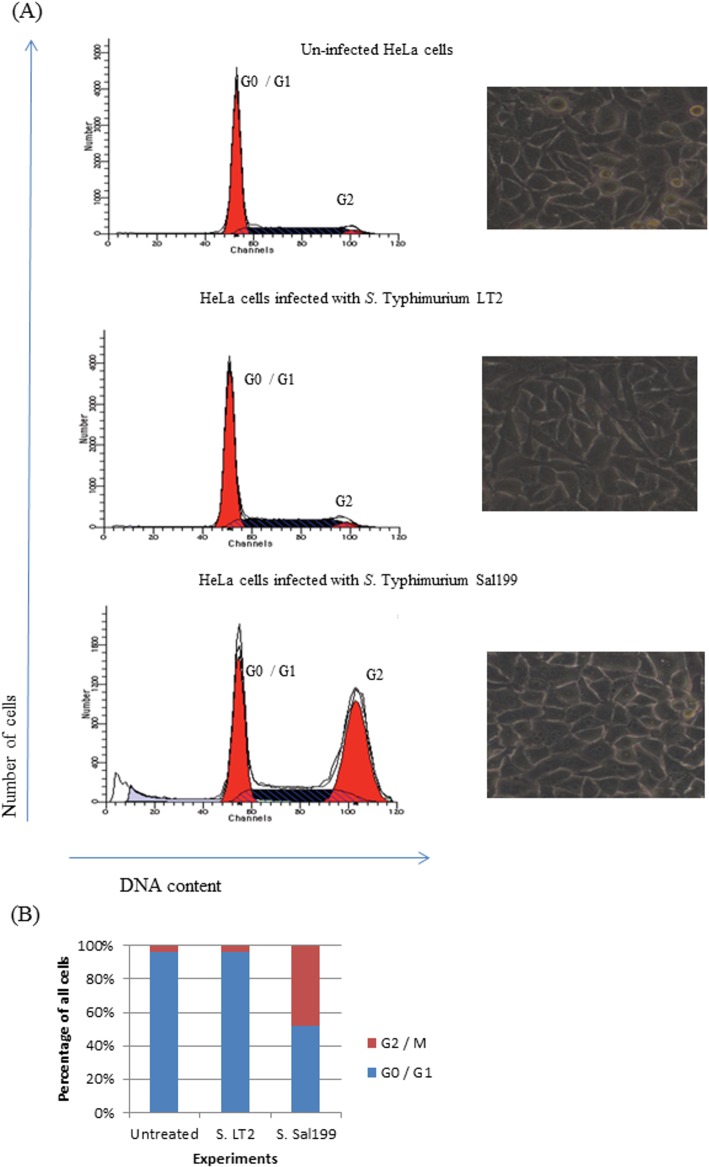
(A) Effect of the CdtB toxin in HeLa cells, 72h after *S*. Typhimurium Sal199 infection. On top, untreated HeLa cells, in middle, HeLa cells infected with *S*. Typhimurium LT2, and on bottom, HeLa cells infected with *S*. Typhimurium Sal199. The cells were examined at same magnification (40x) by light microscope, and the cell cycle arrest measured by flow cytometry. The peaks corresponding to cells in G1, S and G2/M are indicated. (B) Ratio G0/G1/G2/M of cell cycle profiles from at least three independent experiments.

Cytolethal distending intracellular tripartite toxin causes cell arrest in G2 / M transition phase, and consequently a nucleus enlargement and an increase in the amount of DNA can be observed in eukaryotic cells. *S*. Typhimurium Sal199 was examined using flow cytometry for its ability to block cell cycle arrest by analysing the DNA content of HeLa cells. HeLa cells infected with *S*. Typhimurium LT2 were essentially identical to the control (no infected cells), with a large accumulation of cells in the G0 / G1. In contrast, HeLa cells infected with Sal199 become arrested in G2 / M phase after 72 h in comparison to un-infected cells (Fig 2A). The ratio G0 / G1 to G2 / M of cell cycle profiles is shown in Fig 2B. The results from profiles of the DNA content suggest that *S*. Typhimurium Sal199 exhibited G2 / M arrest.

### Adhesion/invasion assays of selected isolates

**Fig 3:**
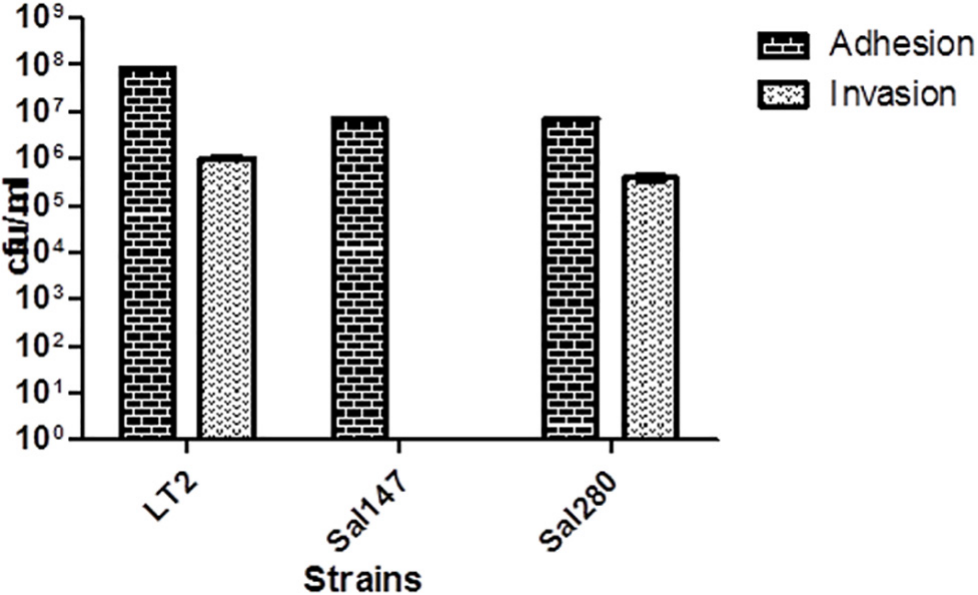
Adhesion and invasion of HT-29 cells by control strain *S*. Typhimurium LT2, *S*. Infantis Sal147, and *S*. Infantis Sal280. Data shown are means ± SEM from four independent experiments.

The arrays and WGS results predicted a difference in virulence between two *S*. Infantis strains tested, therefore we characterised these strains further by adhesion/invasion assays. From microarray and whole genome sequencing, one strain Sal147, has a large deletion in SPI-1 genes while Sal280 harboured all SPI-1 genes. The results of adhesion and invasion assays are shown in Fig 3. There was a statistically significant difference to the extent that *S*. Typhimurium LT2 control strain was able to adhere compared to both *S*. Infantis strains. In addition, *S*. Typhimurium LT2 was 2.6-fold more invasive in HT-29 cells than *S*. Infantis Sal280 (*p* < 0.001). When comparing the two *S*. Infantis strains no statistically significant difference in adhesion was observed, but *S*. Infantis Sal147, was unable to invade HT-29 cells, unlike Sal280 (*p* < 0.001; Fig 3).

### *In vivo* assays

**Fig 4:**
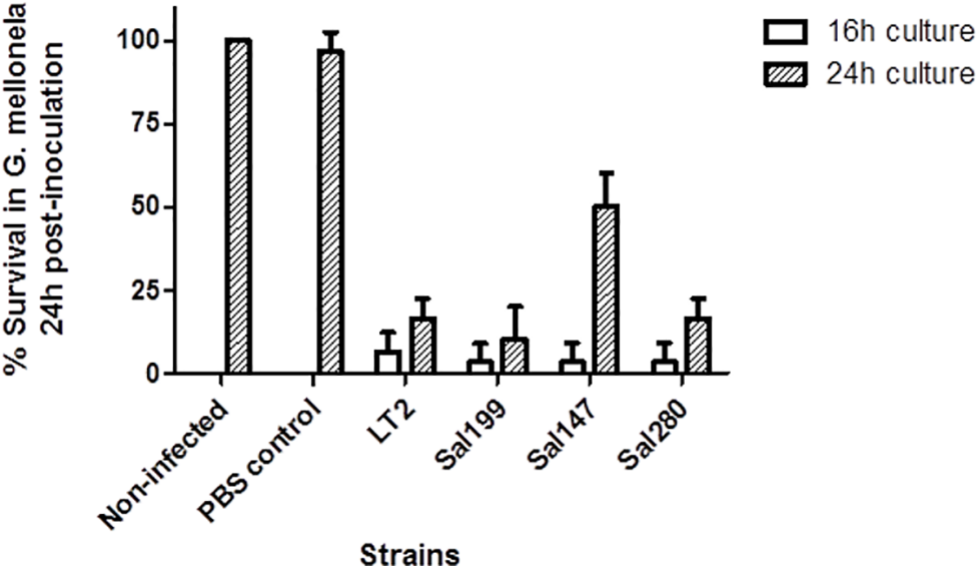
Percentage survival in *G*. *mellonella* 24h post-infection in different strains.

To further assess the virulence potential of *S*. Typhimurium Sal199 and *S*. Infantis Sal147 strains, *in vivo* assays were performed using the larvae of the Greater wax moth *G*. *mellonella*. The mean rate of survival of *Galleria* at this bacterial dose was strain and growth time dependent. Sal147 strain, grown for 24h, showed a higher percentage of survival, at an average of 50% (range 40–60%) than *S*. Typhimurium Sal199 that presented a mean survival of 10% (range 0–20%). *S*. Typhimurium LT2 and *S*. Infantis Sal280 presented a survival rate of 16.7% (range 10–20%) (Fig 4). Therefore, the mean rate of survival for Sal147 infected larvae was up to 3 times higher when compared to Sal280; while the mortality rate of Sal199 infected larvae was 1.6 times higher compared to LT2. A colour change was observed in some larvae following 24 h incubation. Those killed following infection were black or dark brown while surviving larvae were white and looked identical to the uninfected and PBS controls.

## Discussion

Infection with *S*. *enterica* is one of the most important causes of food-borne diseases worldwide. In this study we have developed and tested a high through-put microarray chip for *Salmonella* virulence genes using a collection of *Salmonella* isolates recovered from farm animals, slaughterhouses and retail meat, in order to assess the ability of this array to help identify the potential risk associated with *Salmonella* harbouring different virulence determinants entering the food chain and causing disease in man.

This study showed that adhesion and SPI-located genes were highly conserved in all strains except one *S*. Infantis, while prophages and virulence plasmid were generally variable among the strains and serotypes, as reported previously [9; 14; 27]. Nonetheless, some phage-associated genes were more frequently detected, e.g. *sodCI* (encoding a periplasmatic superoxide dismutase) and *sspH2* (encoding an ubiquitin ligase protein), suggesting its stable maintenance. A core set of fimbrial genes were detected in all strains which could contribute to the colonization in a broad host range; while presence of particular fimbrial genes in *S*. Enteritidis, probably contribute to host specificity [9; 14]. All *S*. Infantis strains were swine-associated and most showed presence of SPI-3 *sugR* gene, contrasting with previous reports [14]. Virulence plasmids are important genetic elements in *Salmonella*. *S*. Typhimurium strains harbouring the *spv* and *rck* genes, which are required for bacterial multiplication in the reticulo-endothelial system are more able to produce systemic disease [28]. These plasmids were mainly present in isolates from the extra-intestinal environment, like liver, muscle, and food products such as raw meat and ready to eat food such as sausages. The *pef* fimbrial operon and *rck* gene that are plasmid borne and play a role in stages of the infection process, were present in all *S*. Enteritidis and in some *S*. Typhimurium isolates allowing their separation into two groups which may correspond to a difference in the potential for these *S*. Typhimurium strains to cause infection and pathogenesis. It also highlights the virulence plasticity of *S*. *enterica*, and its ability to adapt to others hosts. Therefore having a more extensive knowledge of virulence genes will help understands the evolution in different serotypes and to infer their relative virulence. Genome variability is common among *S*. Typhimurium isolates [29] and may be an important factor to consider in relation to virulence genes and consequence of infection. For our panel of isolates ~99% of *S*. Typhimurium strains harboured one or more antibiotic resistance determinant, including to Extended Spectrum β-Lactamases (ESBL), with more than 37.2% showing a multidrug resistance (MDR) phenotype [16].

Moreover, using the virulence arrays we detected a *cdtB* gene, in association with *pltA* and *pltB* genes in *S*. Typhimurium Sal199 strain isolated from raw poultry meat. CdtB forms a tripartite toxin with PltA and PltB, causing cell-cycle arrest in *S*. Typhi, thereby increasing its virulence potential [24]. Our findings are in concordance with previous reports showing the CdtB toxin plays a role in cellular distension of host cells [23; 24]. The *cdtB* gene has been described in only six NTS serotypes from human clinical and poultry origin [29–31], but never in *S*. Typhimurium. This study shows that the HeLa cells infected with *S*. Typhimurium Sal199 became distended with prominent cell cycle arrest compared with the control (un infected cells and cells infected with *S*. Typhimurium LT2). Presence of *cdtB*, *pltA* and *pltB* upstream of an *IS*911 transposase might have resulted in horizontal transfer of these genes from a *S*. Typhi to Sal199, a *S*. Typhimurium strain. To the best of our knowledge, this is the first report detecting the presence of this typhoid-related toxin in *S*. Typhimurium, which is one of the most common serotypes associated with human gastroenteritis. Furthermore, the increased virulence of Sal199 is corroborated by our *in vivo* findings which showed increased mortality of *G*. *mellonella* by *S*. Typhimurium Sal199 with respect to *S*. Typhimurium LT2, which might be associated with expression of the CdtB toxin.

Another interesting finding from using the virulence arrays, and confirmed by WGS, was the absence of thirty-three SPI-1 genes in the MDR *S*. Infantis Sal147. Adhesion/invasion assays showed that this strain was able to adhere but not invade a human colon cell line in comparison to controls. These results indicate a possible reduction in the virulence capacity for Sal147 in comparison to another *S*. Infantis isolate and LT2, which was confirmed using the *G*. *mellonella in vivo* virulence model and suggests a reduced risk to humans.

Therefore, in this study we have clearly shown the benefits of using a virulence genes microarray to assess the risk of infection associated with *Salmonella* strains isolated from farm animals, slaughter houses and food products which can enter the food chain and cause infection in humans. Identification of strains with high virulence potential and infectivity in the food animal breeding section at an early stage may help facilitate interventions that could reduce the risk of dissemination of epidemic strains such as *S*. Enteritidis PT4, *S*. Typhimurium DT104 and monophasic *S*. Typhimurium DT193, which have all entered the food chain to cause human infections. In addition to providing details of the virulence profile of isolates this chip also helped group isolates by serotype, which indicates that isolates of the same serotype usually harbour the same virulence profile, and so this chip can be used as a surrogate for serotyping in future. In addition, it has the benefit of spotting isolates from a serotype with atypical virulence profile which may pose either increased or decreased risk to humans. Currently, these arrays are a cheaper and more accessible option than some of the other methods explored in this study including whole genome sequencing, but this may change in future. Also, they can be used by any laboratory worldwide, as a rapid and useful epidemiological surveillance tool to detect and assess the risk posed to humans by *Salmonella* isolated from the food chain and to help investigate outbreaks.

## Supporting Information

S1 Table Details of the *Salmonella* virulence genes for which probes and labelling primers were designed in this study. (XLS)

S2 Table RT-PCR results to SPI-11 and typhoid-associated genes. (XLSX)

S3 Table Binary form microarrays virulence results. (XLSX)
